# Nitrous oxide production and consumption by marine ammonia-oxidizing archaea under oxygen depletion

**DOI:** 10.3389/fmicb.2024.1410251

**Published:** 2024-09-04

**Authors:** Elisa Hernández-Magaña, Beate Kraft

**Affiliations:** Nordcee, Department of Biology, Faculty of Sciences, University of Southern Denmark, Odense, Denmark

**Keywords:** oxygen depletion, anoxia, NO dismutation, ammonia-oxidizing archaea, nitrous oxide, nitrous oxide reduction

## Abstract

Ammonia-oxidizing archaea (AOA) are key players in the nitrogen cycle and among the most abundant microorganisms in the ocean, thriving even in oxygen-depleted ecosystems. AOA produce the greenhouse gas nitrous oxide (N_2_O) as a byproduct of ammonia oxidation. Additionally, the recent discovery of a nitric oxide dismutation pathway in the AOA isolate *Nitrosopumilus maritimus* points toward other N_2_O production and consumption pathways in AOA. AOA that perform NO dismutation when exposed to oxygen depletion, produce oxygen and dinitrogen as final products. Based on the transient accumulation of N_2_O coupled with oxygen accumulation, N_2_O has been proposed as an intermediate in this novel archaeal pathway. In this study, we spiked N_2_O to oxygen-depleted incubations with pure cultures of two marine AOA isolates that were performing NO dismutation. By using combinations of N compounds with different isotopic signatures (^15^NO_2_^−^ pool +^44^N_2_O spike and ^14^NO_2_^−^ pool +^46^N_2_O spike), we evaluated the N_2_O spike effects on the production of oxygen and the isotopic signature of N_2_ and N_2_O. The experiments confirmed that N_2_O is an intermediate in NO dismutation by AOA, distinguishing it from similar pathways in other microbial clades. Furthermore, we showed that AOA rapidly reduce high concentrations of spiked N_2_O to N_2_. These findings advance our understanding of microbial N_2_O production and consumption in oxygen-depleted settings and highlight AOA as potentially important key players in N_2_O turnover.

## Introduction

Environments with low oxygen concentrations are major sources of the greenhouse gas nitrous oxide (N_2_O). Nearly half of the net yearly production of N_2_O in the open ocean occurs in hypoxic and oxygen-depleted waters (Codispoti, 2010). N_2_O has a warming potential approximately 300 times higher than CO_2_ and contributes to stratospheric ozone destruction (IPCC, 2014). In order to understand the dynamics of N_2_O emissions from oxygen-depleted environments, it is crucial to disentangle the contributions of different microbial pathways of N_2_O production and consumption.

Ammonia-oxidizing archaea (AOA) are key players in the nitrogen cycle, performing the first step of nitrification. They are among the most abundant microorganisms in the ocean, and in some cases, they can represent up to 40% of the total picoplankton in the water column (Karner et al., 2001). Oceanic ammonia oxidation is almost entirely performed by AOA, and they have been suggested to be an important source of N_2_O in the ocean (Santoro et al., 2011; Löscher et al., 2012). Here, N_2_O is mainly formed as a byproduct of ammonia oxidation in a process named hybrid formation. In this process, hydroxylamine from NH_4_^+^ reacts with NO, which is produced from NO_2_^−^ (Stieglmeier et al., 2014; Kozlowski et al., 2016; Prosser et al., 2020; Wu et al., 2020; Stein et al., 2021).

Until the recent discovery of the NO-dismutation pathway in AOA upon oxygen depletion, AOA were assumed to be inactive when oxygen was absent. In this NO-dismutation pathway, AOA reduces NO_2_^−^, which is the product of aerobic ammonia oxidation, to NO. Then, NO is dismutated to O_2_ and N_2_O, which is reduced to N_2_ (Kraft et al., 2022). The dismutation step is thermodynamically favorable (2NO➔N_2_O + 0.5O_2;_ ΔG0’ = −165kJ/mol O_2_), and AOA can use the produced oxygen to fuel ammonia oxidation (Kraft et al., 2022). N_2_O is proposed to be an intermediate based on the transient accumulation of ^15,15^N-labeled N_2_O from ^15^N-nitrite in parallel to oxygen production (Kraft et al., 2022; Hernández-Magaña et al., 2023). NO dismutation has been observed previously in the methane-oxidizing bacterium *Ca. Methylomirabilis oxyfera*, which also produces O_2_ and N_2_ as final products of the pathway (Ettwig et al., 2010). However, there is no evidence of N_2_O production or reduction associated with this process. A further difference is that in the case of *Ca. M. oxyfera*, the oxygen produced is immediately utilized to oxidize methane and other microbial processes (Ettwig et al., 2010). In the case of AOA, the oxygen produced during NO dismutation is used for ammonia oxidation and respiration, but the coupling between production and consumption is not that tight, and oxygen accumulates (Kraft et al., 2022).

AOA are highly abundant in environments with low or undetectable oxygen concentrations, such as anoxic basins such as the Black Sea (Sollai et al., 2019) or oceanic oxygen minimum zones (OMZs) (Francis et al., 2005; Lam et al., 2007; Beman et al., 2008; Peng et al., 2015; Bristow et al., 2016). The discovery of NO dismutation in AOA provides a potential explanation for their presence in these environments, suggesting that AOA may contribute to N_2_O cycling if N_2_O indeed is an intermediate in NO dismutation.

To date, N_2_O production from nitrite in anoxic environments has been solely attributed to denitrification. Denitrification, the stepwise reduction of nitrate to dinitrogen (NO_3_^−^ ➔NO_2_^−^ ➔ NO ➔ N_2_O ➔ N_2_), can be performed by a phylogenetically diverse group of organisms, including bacteria, archaea, and eukaryotes (Thomson et al., 2012). Some denitrifiers possess only some of the enzymes and can only carry out incomplete denitrification; organisms that cannot reduce N_2_O to N_2_ lead to the accumulation of N_2_O (Babbin et al., 2015), while some microorganisms that only reduce N_2_O to N_2_ become net sinks of N_2_O in the system (Jones et al., 2013). Biogeochemical rate measurements based on ^15^N-stable isotope labeling would not be able to distinguish between denitrification and NO dismutation as sources for N_2_O and N_2_ production because, in both processes, the two N atoms originate from nitrite.

To test the role of N_2_O as an intermediate in the NO dismutation pathway by AOA, we carried out incubations under oxygen depletion with pure cultures of the AOA strains, *N. maritimus* and *Nitrosopumilus piranensis*. The oxygen-depleted incubations were combined with the use of ^15^N-stable isotope-labeled compounds to track the origin and fate of the nitrogen gases N_2_O and N_2_ during NO dismutation. The N_2_ and N_2_O accumulation patterns from different experiments support the role of N_2_O as an intermediate in the formation of N_2_ upon oxygen depletion. Furthermore, solid evidence for the N_2_O reduction to N_2_ by two marine AOA isolates is presented.

## Materials and methods

### Growth conditions

Axenic 5 L batch pre-cultures of the AOA strains *N. maritimus* SCM1 and *N. piranensis* D3C (JCM 32271, DSM 106147, and NCIMB 15115) were grown at 28°C in the dark in synthetic Crenarchaeota medium (SCM) HEPES-buffered (pH 7.8), as described by Könneke et al. (2005) and Martens-Habbena et al. (2009), modified with a 6 mM final concentration of sodium bicarbonate (Kraft et al., 2022).

### Oxygen-depleted incubations

The oxygen-depleted incubations were prepared by sparging the aerobically grown batch culture with argon gas (99.99%) for 45 min to reduce the oxygen concentration in the culture. The culture was sterilely transferred into 330-ml custom-made glass bottles designed to avoid oxygen intrusion with a glass capillary and a port for inserting a microsensor (Tiano et al., 2014) through a glass tube connection, using the overpressure generated in the argon-sparged culture bottle. All bottles were filled without headspace and closed with glass stoppers. The bottles were continuously stirred with glass-coated stirring bars (VWR, United Kingdom) at 300 rpm. The bottles were incubated in a water bath at 28°C in the dark. Control incubations with the custom-made bottles and killed controls with HgCl_2_ have been previously reported in Kraft et al. (2022), showing no oxygen intrusion from the atmosphere.

Oxygen was monitored constantly during the incubations with trace fluorescence oxygen sensors, also referred to as optodes, with a detection limit of 0.5 nM (Lehner et al., 2015). The optodes were previously glued to the glass bottles. NO was monitored with microsensors (Unisense, Denmark), inserted into the sensor ports of the bottles, which were previously sterilized with 70% ethanol, and rinsed with autoclaved ASTM1a water. NO was observed to cause a small and predictable interference with the optodes (up to 17%). Therefore, the oxygen concentration measurements were corrected for NO interference, as in Kraft et al. (2022). All bottles, stirring bars, tube connections, and materials used for the incubation were previously autoclaved.

### N_2_O as an intermediate in dinitrogen production via NO dismutation

Two sets of experiments with ^15^N-stable isotope compounds were used for the identification of the intermediates in dinitrogen and oxygen production via NO dismutation.

For the first experimental setup, batch cultures of *N. maritimus* and *N. piranensis* were grown aerobically with ^15^N-labeled ammonium (^15^NH_4_^+^) until it was completely oxidized to ^15^NO_2_^−^, and thus cultures contained a pool of 1 mM of ^15^NO_2_^−^ (late exponential phase). Prior to the incubation, more than 500 μM of ^14^NH_4_^+^ was added to the culture to ensure the survival (ammonia oxidation) of the cultures during the experiment and to capture traces of ^15^NH_4_^+^ that could have remained in a large pool of ^14^NH_4_^+^. The incubations under oxygen depletion were set up, as described in the section “oxygen-depleted incubations.” The sets of replicates (at least 3 bottles of 330 mL each per incubation) were spiked with 1.2 μM of unlabeled N_2_O (^44^N_2_O) after 30 h in the case of *N. maritimus* and with 3 μM after 6 h and 1.2 μM after 30 h in the case of *N. piranensis*. Three incubation replicates were kept without the addition of N_2_O as a control. A killed control was performed by adding mercury chloride to the incubation.

In the second set of experiments, an aerobically grown batch culture of *N. piranensis* was maintained with ^14^NH_4_^+^ until it was completely oxidized to ^14^NO_2_^−^. Then, the batch culture contained a pool of approximately 1 mM of ^14^NO_2_^−^ (the late exponential phase). Prior to the incubation, more than 500 μM of ^14^NH_4_^+^ was added to the culture to ensure the survival (ammonia oxidation) of the cultures during the experiment. The oxygen-depleted incubation was started, as described in the section “oxygen-depleted incubations.” One set of replicates (at least three bottles of 330 mL each) was spiked with 40 nM of ^15^N-labeled N_2_O (^46^N_2_O) at 8 h and with 90 nM at 42 h. Three incubation replicates were kept without the addition of ^46^N_2_O as a control.

### Sample collection and analysis

Samples were collected with gas-tight syringes (Hamilton, United States) that were connected to stainless steel needles (Ochs, Germany) through the capillaries of the incubation bottles. When collecting the samples, the volume collected was simultaneously replaced with deoxygenated sterile culture media to avoid headspace formation in the incubation bottle. The samples were collected in 3-ml gas-tight exetainers, headspace-free, and preserved with 50 μL of saturated HgCl_2_ solution. The isotopic signature of N_2_ and N_2_O was analyzed by coupled gas chromatography–isotope ratio mass spectrometry (GC-IRMS) on a Thermo Delta V Plus isotope ratio mass spectrometer (Dalsgaard et al., 2012). Total N_2_O concentrations were analyzed using a gas chromatograph (GC-TRACE1300, Thermo Scientific) equipped with an electron capture detector. Concentrations were plotted as the average of at least three replicates, with error bars representing the standard deviation. Rates were calculated from the change in concentration over time, with *r*^2^ > 0.9.

## Results

### Reduction of N_2_O to N_2_ by *N. maritimus* and *N. piranensis* under oxygen depletion

The AOA strains *N. maritimus* and *N. piranensis* were previously observed to conduct NO dismutation upon oxygen depletion (Kraft et al., 2022; Hernández-Magaña et al., 2023), in which they produced oxygen and ultimately N_2_ from NO_2_^−^. Transient accumulation of N_2_O in both strains was reported in the cited publications, suggesting that AOA can produce N_2_O under oxygen depletion and further reduce it to N_2_. To assess the role of N_2_O as an intermediate in NO dismutation by AOA and, therefore, the AOA’s potential to reduce N_2_O, we performed incubations under oxygen depletion with pure cultures of *N. maritimus* and *N. piranensis.* The first set of incubations was started with a pool of ^15^NO_2_^−^ and spiked with 1.2–1.5 μM of unlabeled nitrous oxide (^44^N_2_O) at 30 h for both AOA strains and additionally with 3 μM of ^44^N_2_O at 6 h only for *N. piranensis*.

**Figure 1:**
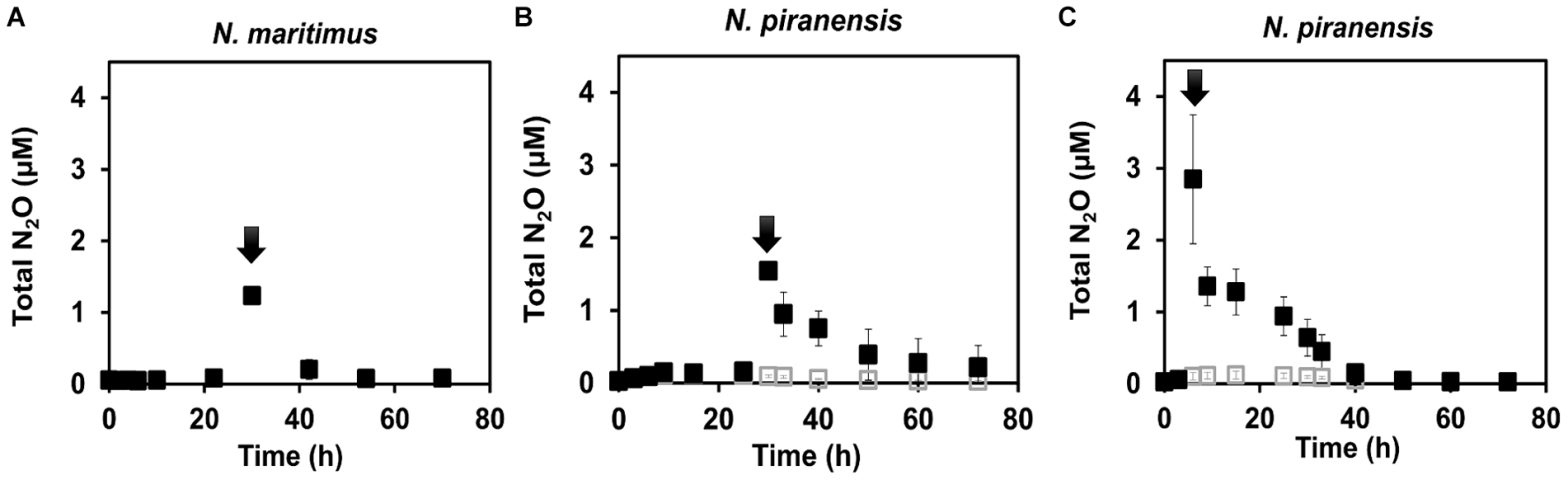
N_2_O consumption in oxygen-depleted incubations of AOA cultures receiving a spike of N_2_O (black arrows). **(A)** *N. maritimus* spiked with 1.2 μM of ^44^N_2_O at 30 h of incubation. (**B)** *N. piranensis* spiked with 1.5 μM of ^44^N_2_O at 30 h of incubation. **(C)** *N. piranensis* spiked with 3 μM of ^44^N_2_O at 6 h. Filled squares show the spiked incubations, while open squares are control replicates (without spike). Symbols represent averages of triplicates, and error bars represent the standard deviation. Some error bars are smaller than the symbols.

**Figure 2:**
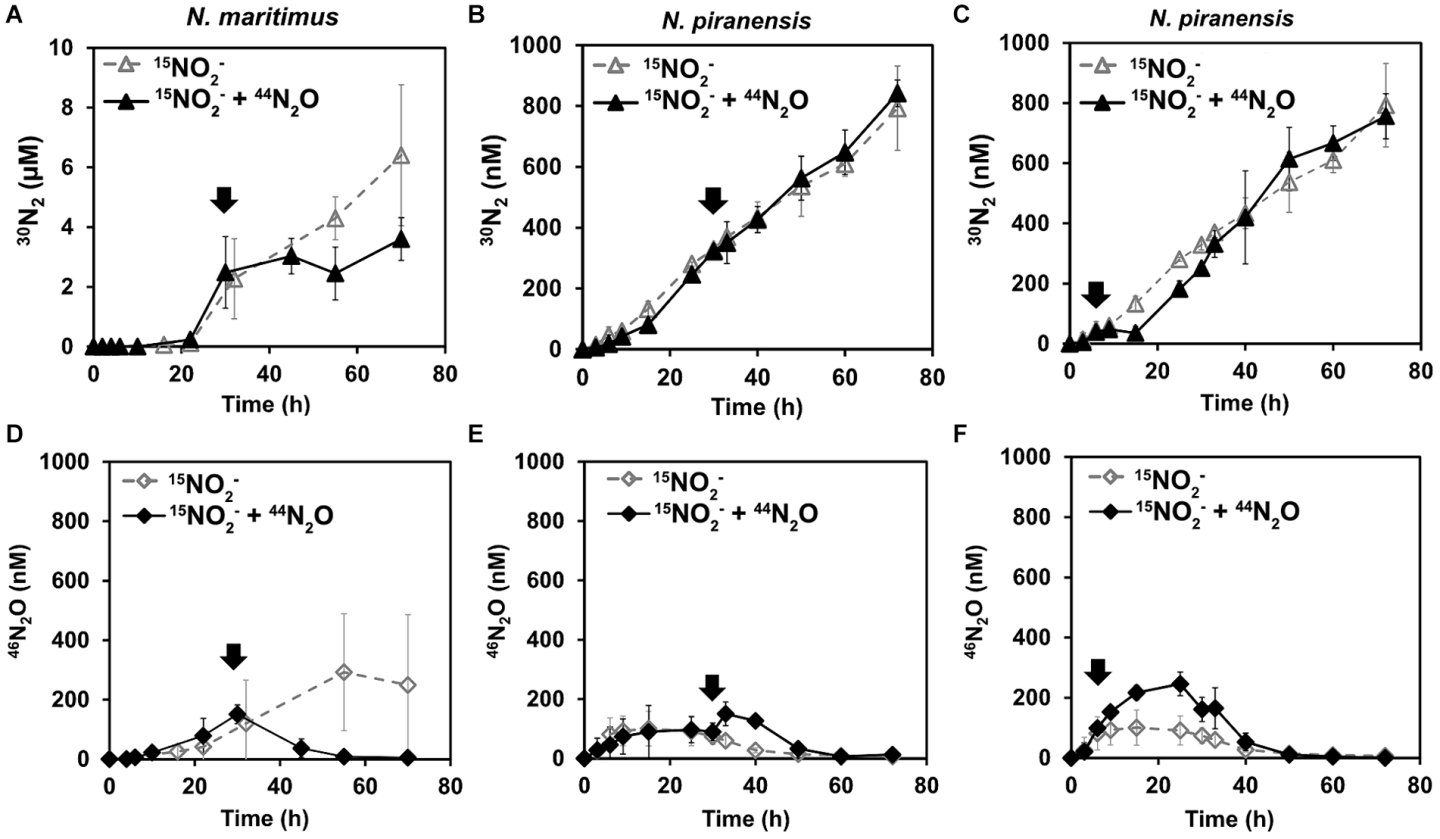
Effect of ^44^N_2_O spikes on ^30^N_2_ and ^46^N_2_O accumulation by AOA under oxygen depletion. All incubations started with a pool of ^15^NO_2_^−^, and ^44^N_2_O was spiked (marked by arrows). The top panels show the accumulation trends of ^30^N_2,_ while the bottom panels show the parallel ^46^N_2_O accumulation for the same set of incubations: **(A,D)** from *N. maritimus* with ^44^N_2_O spiked at 30 h **(B,E)** from *N. piranensis* with ^44^N_2_O spiked at 30 h, and **(C,F)** from *N. piranensis* with ^44^N_2_O spiked at 6 h. Open symbols represent control incubations (only ^15^NO_2_^−^ pool), while black symbols show the spiked treatment. The average values of at least three replicates are presented; error bars represent the standard deviation. Some error bars are smaller than the symbols and are therefore not visible.

A striking decrease in the total N_2_O concentration was observed after the spike in all the incubations. N_2_O consumption was especially fast within the first 3 h after the spike (Figure 1). For example, *N. piranensis* consumed on average 497 nM/h in the first 3 h after the 6-h spike and 198 nM/h after the 30-h spike. Overall, strikingly fast N_2_O consumption after the spikes was consistently observed in all the incubations. After this first fast decrease in N_2_O, N_2_O consumption slowed down. Then, *N. maritimus* had the highest consumption rate of 48 nM/h, followed by the incubation of N. piranensis after the spike at 6 h, which had a rate of 41 nM/h. Finally, the same strain after the spike at 30 h consumed all spiked N_2_O in approximately 30 h at a rate of 28 nM/h. The accumulation of ^46^N_2_O from ^15^NO_2_^−^ started within the first hours of the oxygen-depleted incubations, followed by a linear production of ^30^N_2_ (Figure 2, controls). In *N. maritimus* incubations, the production of ^30^N_2_ increased at approximately 20 h, while in *N. piranensis* N_2_ production was linear from the beginning of the oxygen-depleted incubation. Another subtle difference between the strains was the transient accumulation of N_2_O, which was maintained throughout the whole incubation period for *N. maritimus.* For *N. piranensis,* the N_2_O accumulation started quickly after oxygen depletion, reaching its maximum within the first 20 h and decreasing almost totally after 40 h of oxygen depletion. Despite these differences in accumulation patterns between strains, there is consistency in the transient accumulation of ^46^N_2_O and in the formation of ^46^N_2_O only from ^15^NO_2_^−^ via NO, which is consistent with previous observations (Kraft et al., 2022; Hernández-Magaña et al., 2023).

If N_2_O is a free intermediate in the NO-dismutation pathway (a product of the NO-dismutation step), which is reduced to N_2_ and not a byproduct, an increase in the pool of ^44^N_2_O over ^46^N_2_O (^44^N_2_O spike) would lead to an increase in ^28^N_2_ production instead of ^30^N_2_ production compared to the control incubations, in which only ^46^N_2_O is available. Thus, the reduction of N_2_O from a pool enriched with ^44^N_2_O would be observed as a slowing of ^30^N_2_ accumulation. For the incubation with *N. maritimus* in which ^44^N_2_O was spiked at 30 h (Figure 1A), ^30^N_2_ accumulation stopped until the spiked ^44^N_2_O was consumed (55 h) and then ^30^N_2_ accumulation started again (Figure 2A), demonstrating the direct reduction of N_2_O to N_2_ and the role of N_2_O as an intermediate in the NO-dismutation pathway. For the incubations with *N. piranensis,* a similar pattern in ^30^N_2_ production was observed after the spike of ^44^N_2_O at 6 h of the incubation (Figure 2C). In the case of the spiked incubations of *N. piranensis* at 30 h, the effect of the ^44^N_2_O spike on the ^30^N_2_ production was more difficult to notice in the averaged trend (Figure 2B) and easier to distinguish in the trends of the individual replicates (Supplementary Figure S1A). The replicate with the fastest total N_2_O consumption (Supplementary Figure S1B) was the only replicate with no visible effect on the ^30^N_2_ production after the 30-h spike (Supplementary Figure S1A), suggesting that the N_2_O pool was consumed too fast to capture the N_2_O produced from nitrite. Additionally, no production of ^30^N_2_ or ^46^N_2_O or consumption of N_2_O after a spike of ^44^N_2_O was detected in the killed control with *N. maritimus* (Supplementary Figure S2), indicating that the consumption of N_2_O was performed by active cells of AOA.

**Figure 3:**
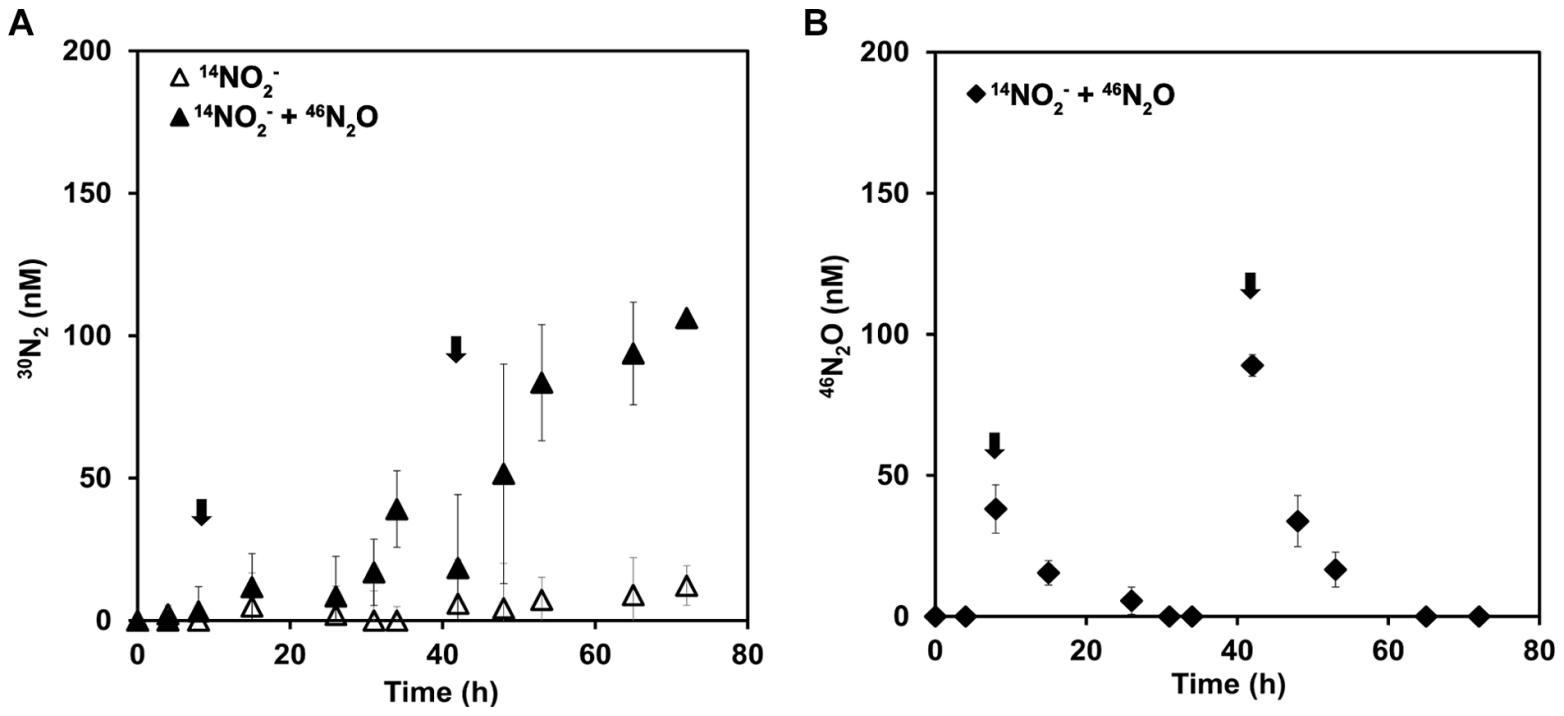
^46^N_2_O turnover in oxygen-depleted incubation of *N. piranensis* under oxygen depletion. Incubations started with a pool of ^14^NO_2_^−^ and ^46^N_2_O was spiked at 6 h and 42 h (black arrows). **(A)** ^30^N_2_ production. Black triangles represent incubations into which ^46^N_2_O was spiked; open triangles indicate controls without spikes. **(B)** ^46^N_2_O concentration measured in the incubation. Black diamonds represent the incubations in which ^46^N_2_O was added. ^46^N_2_O was undetectable in the control replicates; thus, symbols are not presented. The average of at least three replicates is presented; error bars represent the standard deviation. Some error bars are smaller than the symbols and therefore not visible.

Complementary incubations to the previous ones were performed to further explore the ability of AOA to reduce N_2_O to dinitrogen. *N. piranensis* was selected based on the observations in previous incubations that pointed toward a faster N_2_O turnover during NO dismutation. For these incubations, the concentration of spiked N_2_O was reduced so that the total N_2_O concentration remained in the range in which *N. piranensis* was previously observed to accumulate, to better simulate the conditions under which the reduction of N_2_O to N_2_ naturally takes place. To track the outcome of the small spikes of N_2_O in this set of incubations, the ^15^N-labeled compound was N_2_O and not nitrite. Oxygen-depleted incubations were performed with a batch culture with a pool of ^14^NO_2_^−^. After 8 h, 40 nM of ^46^N_2_O was spiked into the incubation bottles. The added ^46^N_2_O was completely consumed approximately 24 h after the spike at a rate of approximately 1.5 nM/h (Figure 3A). After 42 h of incubation, a second addition of ^46^N_2_O was made, this time aiming for a final concentration of approximately 90 nM. The ^46^N_2_O added was rapidly consumed again the second time at a rate of approximately 3.5 nM/h.

^30^N_2_ production was only observed in the replicates spiked with ^46^N_2_O (Figure 3B) and was within the expected range. After the second spike, up to 106 ± 2 nM of ^30^N_2_ was produced by the end of the incubation (Figure 3B), indicating a complete conversion of the spiked ^46^N_2_O to N_2_. The measured ^46^N_2_O spike was 38 ± 9 nM in the first spike and 89 ± 4 nM in the second spike. Thus, the incubation received a total of 127 ± 9 nM ^46^N_2_O. Every time a sample was collected, the volume was replaced with anoxic sterile medium (see Materials and Methods), leading to a dilution of the added ^46^N_2_O and the produced ^30^N_2_. Taking this into account, the expected concentration of ^15-15^N compounds at the end of the incubation was 106 nM (see Supplementary material), consistent with the ^30^N_2_ accumulated by the end of the incubation. To summarize, the spiked ^46^N_2_O was completely reduced to and recovered as ^30^N_2._

In the controls that did not receive any ^15^N-labeled compounds, accumulation of neither ^30^N_2_ nor ^46^N_2_O was observed. In these incubations, the accumulation of unlabeled N_2_O started in all replicates at the beginning of the incubation and continued until the spike. After the ^46^N_2_O spike, the total N_2_O concentration (^44^N_2_O + ^46^N_2_O) was slightly higher in the spiked replicates compared to the controls. Overall, the total N_2_O concentration remained within the natural range in which *N. piranensis* would normally accumulate (Supplementary Figure S3). Taken together, the results of both sets of incubations present solid evidence for N_2_O turnover by AOA under oxygen depletion and support its role as an intermediate in the NO-dismutation metabolic pathway.

### Oxygen accumulation dynamics in *N. maritimus* and *N. piranensis*

**Figure 4:**
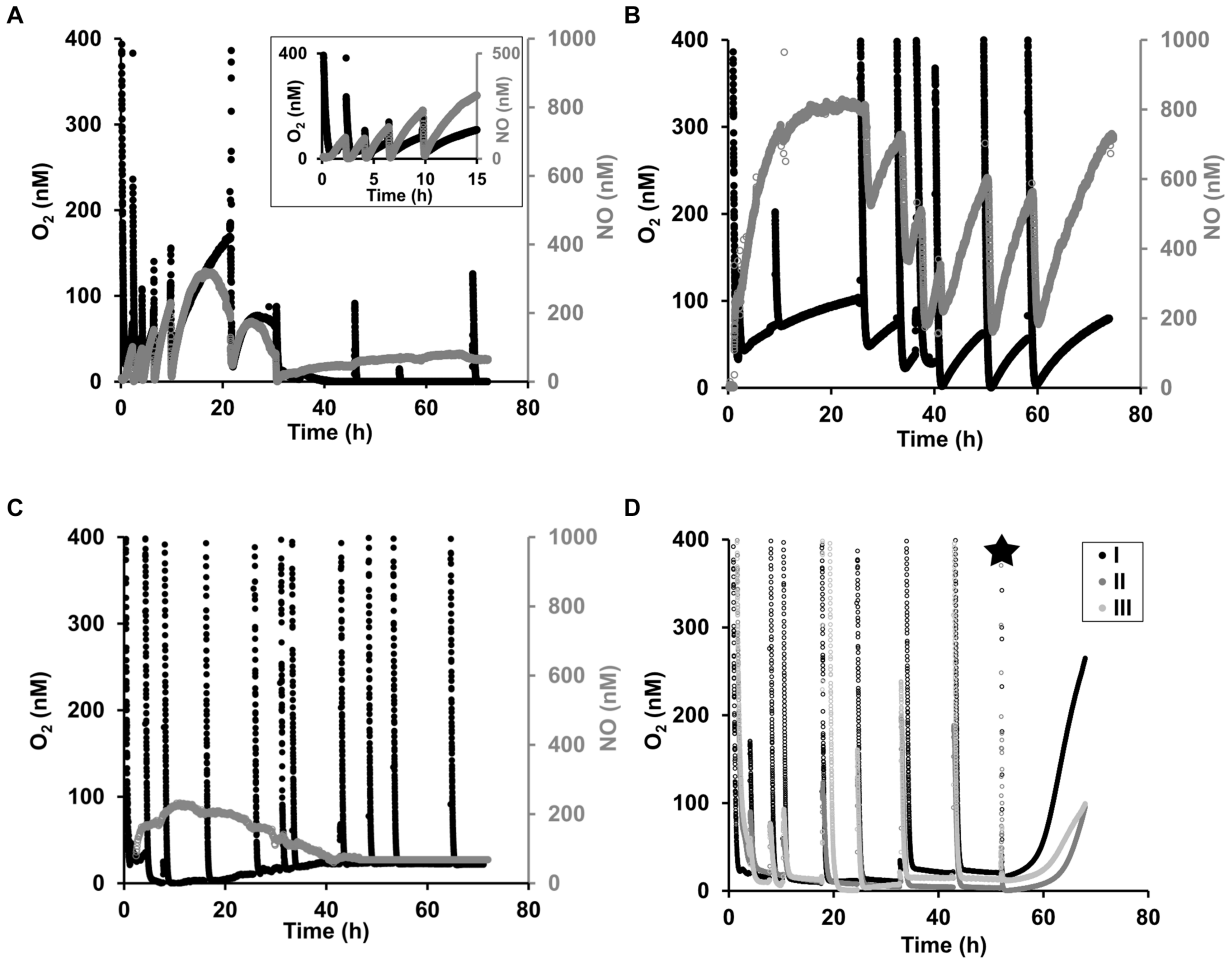
Oxygen dynamics in the nanomolar range in oxygen-depleted incubations for *N. maritimus* and *N. piranensis.* **(A)** Oxygen accumulation (black) and NO accumulation (gray) by *N. maritimus* under oxygen depletion. This example of oxygen accumulation corresponds to the incubations of *N. maritimus* started with a ^15^NO_2_^−^ pool and a spike of ^44^N_2_O at 30 h (Figures 1, 2). One out of three reproducible replicates is shown, and the other replicates are shown in Supplementary Figure S4, S5. **(B)** *N. piranensis* accumulated oxygen (black) and NO (gray) in different batch incubations. One out of three reproducible replicates is shown here, and the other replicates are shown in Supplementary Figure S6. This incubation was not used for the N_2_O spike experiments reported in the present study, but the starting conditions were the same (NO_2_^−^ pool and oxygen depletion). In some batches of *N. piranensis*, oxygen was quickly respired but not accumulated after depletion. **(C)** Shows an example of this. Transient N_2_O accumulation and N_2_ production were observed in parallel (Figures 1, 2, *N. piranensis*). The example here corresponds to the control incubations with ^14^NO_2_^−^ without spike (The other replicates are shown in Supplementary Figure S7A). The spike of ^46^N_2_O at 8 h and at 42 h (Supplementary Figure S6B) did not show differences in oxygen trends. **(D)** After KCN addition (black star), oxygen builds up in incubations with *N. piranensis.* The example here corresponds to the control of the incubations with ^15^NO_2_^−^ without spike. No differences in oxygen trends between control and N_2_O spiked replicates were observed (Supplementary Figure S8). Transient N_2_O accumulation and N_2_ production were observed in parallel (*N. piranensis*, Figures 1, 2).

In order to resolve trends in oxygen consumption and accumulation, oxygen concentrations were measured during all incubations with sensors that can resolve oxygen concentrations in the nanomolar range (Lehner et al., 2015). In all incubations, the oxygen was respired within the first few minutes after the transfer to the incubation bottles. For *N. maritimus*, shortly after the oxygen was depleted, oxygen started to accumulate, coupled with NO accumulation (Figure 4A). This pattern of oxygen accumulation has been reported previously by Kraft et al. (2022) for *N. maritimus* and for other AOA species, including *N. piranensis*, by Hernández-Magaña et al. (2023). When samples were collected, despite the precautions taken (see methodology), the sampling was always accompanied by a small intrusion of oxygen, hereafter referred to as oxygen pulses. Immediately after the oxygen pulses caused by the sampling, oxygen was respired until depletion and oxygen accumulation started again, which was consistently within the nanomolar range and coupled with the transient N_2_O accumulation and N_2_ production described in the previous section.

In addition to the overall oxygen trends described, there was some variability between strains and among incubations. In the incubations with *N. maritimus,* sometimes there was a decrease in oxygen accumulation toward the end of the incubations (Figure 4A and Supplementary Figure S4A), as also observed by Kraft et al. (2022). NO accumulated coupled with oxygen accumulation, especially within the first 20 h of the incubation, reached its highest concentration during this time. Like oxygen, NO accumulation decreased toward the end of the incubation (Supplementary Figure S4B). Oxygen was still consumed following oxygen pulses, indicating the culture’s activity. The cessation of oxygen accumulation should not necessarily be interpreted as a lack of oxygen production, but most likely as a more efficient use of it, as in the case of the methane oxidizer *Ca*. *Methylomirabilis oxyfera*, which internally consumes all the oxygen produced via NO dismutation (Ettwig et al., 2010).

In the case of *N. piranensis,* variability in oxygen accumulation trends between different culture batches was observed. In some incubations, oxygen accumulated after its consumption (Figure 4B), but in other cases, no oxygen accumulation was observed (Figures 4C,D). However, NO, N_2_O, and N_2_ accumulation from NO_2_ were still observed during these incubations. A possible explanation for the lack of oxygen accumulation while the production of N_2_ continued is that NO dismutation continued and the produced oxygen was used more efficiently, as mentioned above. A possible contamination of the culture, which could also lead to the consumption of oxygen produced during incubation, was excluded by fluorescence microscopy.

To test whether *N. piranensis* consumed oxygen more efficiently and thus prevented its accumulation, in some sets of incubations, 0.5 mM of potassium cyanide was added to inhibit oxygen respiration by heme–copper oxygen reductases (Wilson et al., 1994). Indeed, after cyanide addition, oxygen accumulated rapidly (Figure 4D and Supplementary Figure S8A), confirming that oxygen was still being produced but was consumed directly, preventing the accumulation of detectable oxygen concentrations.

## Discussion

### Marine ammonia-oxidizing archaea reduce N_2_O to dinitrogen under oxygen depletion

Oxygen and dinitrogen production through NO dismutation has been observed so far in several different marine and terrestrial AOA isolates, including *N. maritimus* (Kraft et al., 2022) and *N. piranensis* (Hernández-Magaña et al., 2023), which we selected to study the pathway in more detail. In the proposed NO-dismutation pathway, the product of ammonia oxidation, NO_2_^−^, is reduced to NO, which is dismutated. The proposed products of the dismutation step are N_2_O and O_2_. N_2_O is then further reduced to N_2_, making it an intermediate in the NO-dismutation pathway. Transient N_2_O accumulation was observed from the beginning of the incubations, followed by N_2_ accumulation when *N. maritimus* and *N. piranensis* were exposed to oxygen depletion (this study, Kraft et al., 2022; Hernández-Magaña et al., 2023). The production of N_2_O and oxygen from NO dismutation by AOA is a substantial difference from the other known NO-dismutation metabolism by *Ca. M. oxyfera*. This bacterium directly produces N_2_ and oxygen via NO dismutation without N_2_O as an intermediate (Ettwig et al., 2010). By using a combination of oxygen-depleted incubations with different ^15^N-labeled compounds and N_2_O spikes with different isotopic signatures, we confirmed that N_2_O is the direct intermediate of NO dismutation by AOA. Furthermore, the investigated AOA isolates not only turn over N_2_O from NO dismutation but rapidly reduce externally supplied N_2_O to N_2_.

Although the production and accumulation of N_2_O by AOA had been previously reported (Santoro et al., 2011; Löscher et al., 2012), they had been mainly attributed to hybrid formation from ammonia oxidation products and nitrite in oxic incubations (Stieglmeier et al., 2014; Kozlowski et al., 2016; Hink et al., 2017). The N_2_O produced in the mentioned studies showed a hybrid isotopic signature, suggesting that one of the N atoms originated from hydroxylamine and one from NO_2_. A recent study by Wan et al. (2023), using dual-isotope labeling, assessed multiple N_2_O formation mechanisms by *N. maritimus* and suggested ammonia as the main source of N atoms in N_2_O under oxic conditions. The same study also found that the production of N_2_O from nitrite only occurred by hybrid formation when ammonia and oxygen were present. Under the oxygen concentrations used by Wan et al. (2023), the ^46^N_2_O formation from ^15^NO_2_^−^ was negligible, and the authors suggested that the production of N_2_O by NO dismutation in AOA is restricted to anoxia. In our experiments, the isotopic signature of the N_2_O accumulated by *N. maritimus* and *N. piranensis* upon oxygen depletion (^46^N_2_O) indicates that the origin of both N atoms in N_2_O is the pool of ^15^NO_2_^−^, suggesting that under oxygen depletion, NO_2_^−^ is the only source of N atoms for N_2_O formation, which is consistent with the observations by Kraft et al. (2022). It is worth highlighting that oxygen depletion is required for NO accumulation and, consequently, for NO dismutation to take place. At higher oxygen concentrations, NO would not accumulate to the concentrations observed in the incubations presented here because it reacts with oxygen via autooxidation, producing NO_2_^−^ (Ford et al., 1993; Hickok et al., 2013).

During the incubations with a pool of ^15^NO_2_^−^, *N. maritimus* and *N. piranensis* accumulated oxygen, NO, and ^46^N_2_O and produced ^30^N_2_. In the incubations of *N. piranensis* with a pool of ^14^NO_2_^−^, the spiked ^46^N_2_O resulted in the accumulation of ^30^N_2_, with N_2_O being the only source of ^15^N atoms to form dinitrogen. The quick consumption of the spiked N_2_O shows that *N. maritimus* and *N. piranensis* quickly turn over the N_2_O pool to N_2_ when exposed to oxygen depletion. This evidence supports the role of N_2_O as an intermediate in the NO-dismutation pathway. To the best of our knowledge, this is the first time that direct N_2_O reduction to dinitrogen by AOA has been shown in physiology experiments.

When exposed to anoxia, NO dismutation is advantageous for AOA because it constitutes an alternative pathway to sustain energy generation and provides alternative electron acceptors and oxygen that can sustain ammonia oxidation at nanomolar ranges (Kraft et al., 2022). While the NO-dismutation reaction is electron-neutral, the other N-conversion steps in the pathway require electrons. The reduction of NO_2_^−^ to NO requires one electron per molecule of NO produced, and the reduction of N_2_O to N_2_ requires two electrons per molecule of N_2_ produced. While the electrons could be partly supplied by ammonia oxidation, the source of the remaining electrons has yet to be discovered. Potential electron donors are organic compounds naturally accumulated in the culture medium during aerobic cell growth (Bayer et al., 2019, 2022).

If AOA were capable of using alternative electron donors other than ammonia, N_2_O could serve as the sole electron acceptor under anoxia. The rapid conversion of N_2_O to N_2_ in the two AOA isolates investigated here supports this possibility. Metabolic activity and growth with N_2_O as the only electron acceptor are common in many different denitrifying and non-denitrifying microorganisms, with a NosZ N_2_O reductase (Mania et al., 2016; Conthe et al., 2018; Lycus et al., 2018; Read-Daily et al., 2022). Given the incubation times in the present study, cell growth was not expected to be observed. During aerobic ammonia oxidation under oxic and optimal conditions, AOA grow at a relatively slow rate. Generation times of *N. maritimus* and *N. piranensis* are at a minimum of 19 and 27 h, respectively (Qin et al., 2017; Bayer et al., 2019). Growth rates under anoxic conditions are expected to drop. Therefore, to detect cell growth when AOA perform NO dismutation under oxygen depletion, future research should explore alternatives to cell counts, such as using activity proxies like the incorporation of specifically labeled substrates (Musat et al., 2012; Hatzenpichler et al., 2020).

Although nitrite reduction to NO is most likely performed by the NirK nitrite reductase (Bartossek et al., 2010; Kozlowski et al., 2016), the enzymes responsible for NO dismutation and the further reduction of N_2_O by AOA remain to be identified. No genes encoding potential NO dismutases or N_2_O reductases have been identified in the genomes of *N. maritimus*, *N. piranensis,* or other AOA species (Walker et al., 2010; Qin et al., 2020). All known nitrous oxide reductases belong to the NosZ family. However, the existence of N_2_O reductases outside of this family has been proposed multiple times, as reduction of N_2_O has been observed in pure microbial cultures that lack a NosZ enzyme (Arciero et al., 2002; Fernandes et al., 2010). Recently, the cytochrome P450 was suggested to be involved in the production of N_2_O via NO reduction by the AOA *Nitrosocosmicus oleophilus* MY3, based on N_2_O production measurements coinciding with higher expression of the cytochrome (Jung et al., 2019). These observations were made under oxic conditions and low pH (5.5), in contrast to the conditions used in the current study (oxygen depletion and media HEPES buffered at a pH of 7.6). In *Ca. M. oxyfera*, quinol-dependent NO reductases (qNORs) have been identified as putative NO dismutases encoded by the *nod* gene (Ettwig et al., 2010, 2012; Zhu et al., 2019), but AOA do not possess these genes. To date, the potential for NO dismutation followed by N_2_O reduction to N_2_ by AOA would therefore be overlooked in comparative genomic analyses.

In physiological studies, NO dismutation by AOA would also have been easily overlooked because AOA cultures were not exposed long enough to oxygen depletion, and nitrogen compounds were studied with lower-resolution methods. The lowest oxygen concentrations examined in previous physiological studies of AOA were approximately 1 μM in the headspace (0.1%) (Qin et al., 2015, 2017), and ammonia oxidation was no longer detectable with colorimetric assays. These oxygen concentrations greatly exceed the concentrations at which we observed NO dismutation and oxygen accumulation. At the oxygen concentrations of the present study, the ammonia oxidation rates are in the range of 40 nM/h and would only be detectable by using ^15^N-tracers, as shown by Kraft et al. (2022). Furthermore, the rates of N_2_ production via NO dismutation are also low and would not be detectable without the use of ^15^N-tracers.

### Variability in oxygen accumulation trends in marine AOA

In oxygen-depleted incubations in which oxygen accumulation was observed in all replicates, oxygen accumulation often decreased toward the end of the incubation, similar to previous observations (Kraft et al., 2022; Hernández-Magaña et al., 2023). The quick oxygen respiration after oxygen pulses, despite oxygen not being accumulated, indicates respiratory activity of the AOA cells (Figure 4). Moreover, the reduction of N_2_O and constant production of N_2_ in these incubations continued despite the apparent lack of oxygen accumulation. In the specific case of *N. piranensis,* oxygen did not accumulate in some incubation bottles (Figures 4C,D). After cyanide addition, a rapid increase in oxygen concentration was observed (Figure 4D and Supplementary Figure S7A). These observations, taken together, suggest that the cultures most likely continued to produce oxygen via NO dismutation, but all of it was utilized immediately, leading to no accumulation at detectable concentrations. Therefore, the lack of oxygen accumulation does not imply a lack of activity or absence of oxygen production, but most likely a more efficient usage of the oxygen produced, which prevents accumulation from being detected.

These observations point toward a change in the efficiency of the coupling between oxygen production and its use: at the beginning of the incubation, oxygen is produced faster than it is used, and later the production and consumption processes become more tightly coupled, or in the case of *N. piranensis*, some culture batches may have a faster response to oxygen depletion, which leads to a tighter coupling between oxygen production and consumption. Tightly coupled oxygen production and consumption takes place in cultures of the methane oxidizer *Ca. M. oxyfera*: no oxygen accumulates during NO dismutation, as it is immediately utilized intracellularly for methane oxidation (Ettwig et al., 2010). In *Ca. M. oxyfera*, the detection of oxygen produced via NO dismutation was only possible after the inhibition of the oxygen-consuming methane mono-oxygenase complex (pMMO) by acetylene (Ettwig et al., 2010, 2012; Wu et al., 2011), which is comparable to our observation of oxygen accumulation after cyanide addition.

Whether AOA are capable of producing oxygen via the NO-dismutation pathway in the environment is still unknown and challenging to detect, as any trace of oxygen produced in the environment would be immediately used by the microbial community (Garcia-Robledo et al., 2017), and because the isotopic signature of ^15^N-tracer methods to detect NO dismutation is indistinguishable from denitrification. Therefore, it is important to perform investigations in environmental settings to unveil the potential influence of AOA activity on the oxygen and nitrogen metabolism of natural communities in oxygen-depleted ecosystems.

## Conclusion

We confirmed that in the NO-dismutation pathway performed by AOA under oxygen depletion, N_2_O is indeed an intermediate and demonstrated that NO is dismutated to oxygen and nitrous oxide, which is then further reduced to dinitrogen. Through incubations with combinations of different N compounds with different isotopic signatures (^15^NO_2_^−^ pool +^44^N_2_O spike and ^14^NO_2_^−^ pool +^46^N_2_O spike), we showed that N_2_O is rapidly turned over by AOA and that AOA are capable of reducing N_2_O to N_2_ at high rates. The observations made here highlight the importance of a new pathway of N_2_O turnover by AOA, whose potential in the environment needs to be further investigated. AOA have been shown to be abundant in environments with short or extended periods of anoxia, such as marine OMZs or anoxic basins. Experimental evidence of AOA activity at such sites is crucial to determining the extent to which this pathway should be included among the potential sources and sinks of N_2_O in the environment.

## Data Availability

The original contributions presented in the study are included in the article/Supplementary material, further inquiries can be directed to the corresponding authors.
