# Corrigendum: Nitrous oxide production and consumption by marine ammonia-oxidizing archaea under oxygen depletion

**DOI:** 10.3389/fmicb.2024.1506979

**Published:** 2024-10-28

**Authors:** Elisa Hernández-Magaña, Beate Kraft

**Affiliations:** Nordcee, Department of Biology, Faculty of Sciences, University of Southern Denmark, Odense, Denmark

**Keywords:** oxygen depletion, anoxia, NO dismutation, ammonia-oxidizing archaea, nitrous oxide, nitrous oxide reduction

In the published article, there was an error in affiliation 1. Instead of “Nordcee, Department of Biology, Faculty of Sciences, Nordcee, University of Southern Denmark, Odense, Denmark”, it should be “Nordcee, Department of Biology, Faculty of Sciences, University of Southern Denmark, Odense, Denmark”.

In the published article, there was an error. The name of a microbial isolate was spelled incorrectly.

A correction has been made to **Materials and methods**, *Growth conditions*. This sentence previously stated:

“Axenic 5 L batch pre-cultures of the AOA strains *N. maritimus* SCM1 and *N. piranensis* DC3 (JCM 32271, DSM 106147, and NCIMB 15115) were grown at 28°C in the dark in synthetic Crenarchaeota medium (SCM) HEPES-buffered (pH 7.8), as described by Könneke et al. (2005) and Martens-Habbena et al. (2009), modified with a 6 mM final concentration of sodium bicarbonate (Kraft et al., 2022).”

The corrected sentence appears below:

“Axenic 5 L batch pre-cultures of the AOA strains *N. maritimus* SCM1 and *N. piranensis* D3C (JCM 32271, DSM 106147, and NCIMB 15115) were grown at 28°C in the dark in synthetic Crenarchaeota medium (SCM) HEPES-buffered (pH 7.8), as described by Könneke et al. (2005) and Martens-Habbena et al. (2009), modified with a 6 mM final concentration of sodium bicarbonate (Kraft et al., 2022).”

The authors apologize for this error and state that this does not change the scientific conclusions of the article in any way. The original article has been updated.

