# Supplementary material for: Nitrous oxide production and consumption by marine ammonia-oxidizing archaea under oxygen depletion
Source: Front Microbiol. 2024 Sep 4;15:1410251. doi: 10.3389/fmicb.2024.1410251 (PMC11408285; doi:10.3389/fmicb.2024.1410251)
Supplement: Supplementary file 1 [file Data_Sheet_1.pdf]

Supplementary Material

Nitrous oxide production and consumption by marine ammonia-oxidizing archaea under oxygen depletion.

A. Elisa Hernández-Magaña [1*], Beate Kraft [1*]

[1]Nordcee, Department of Biology, Faculty of Sciences, University of Southern Denmark, Denmark.

*** Correspondence:**
Hernández-Magaña Elisa
elisa@biology.sdu.dk

Beate Kraft
bkraft@biology.sdu.dk

Supplementary text

$^{30}N_2$ production from $^{46}N_2O$ spikes (incubations in Figure 3)

During the set of incubations in which ^{46}N2O was spiked, the measured $^{46}N_2O$ concentration after the spike was 38±9 nM at 8 h and 89±4 nM at 42 h. Thus, the total amount of $^{46}N_2O$ added to the incubation was 127±9 nM. During sample collection, the volume collected, 5 ml per sampling point, from each capillary bottle was replaced with anoxic medium to avoid headspace formation. In total, 60 ml of culture were exchanged for medium in an incubation volume of 330 ml (dilution factor = 1.2), resulting in a dilution of the $^{15\text{-}15}$N compounds in the incubation bottles. Therefore, if the measured 127 nM of $^{46}N_2O$ added in total to the incubations is divided by the dilution factor, we obtain 105.9 nM, which is in line with the 106±2 nM $^{30}N_2$ measured at the end of the incubation. Therefore, the spiked $^{46}N_2O$ was completely recovered as $^{30}N_2$.

Supplementary figures

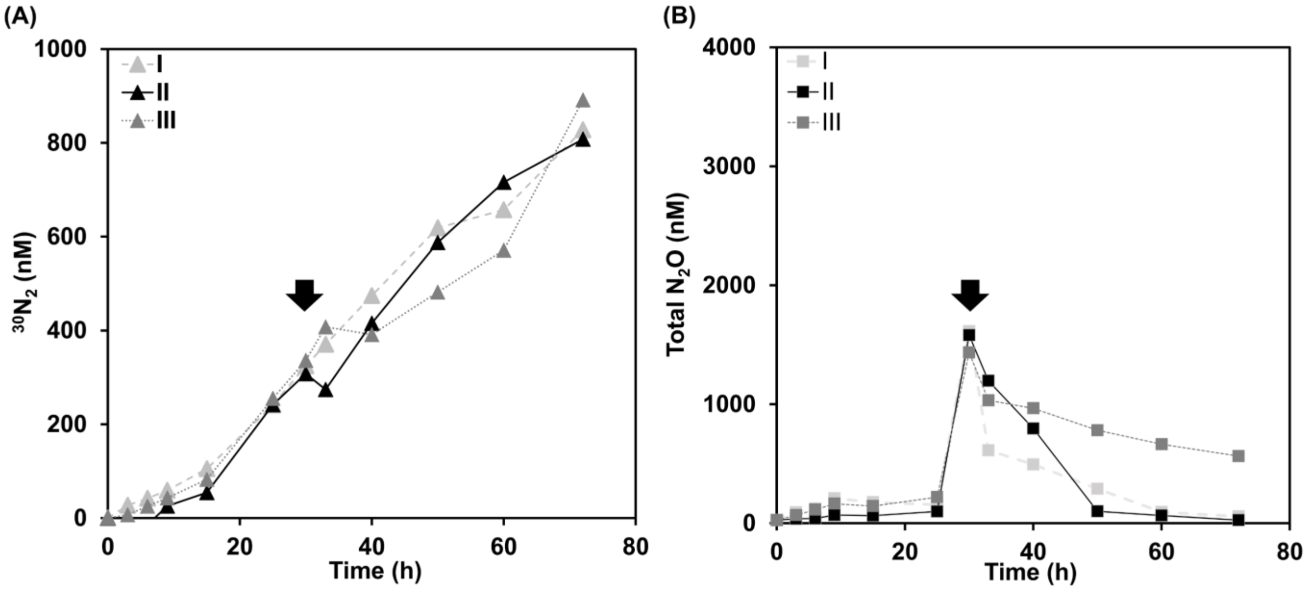

Figure S1. Effect of $^{44}N_2O$ spike on $^{30}N_2$ accumulation **(A)** and total N_2O **(B)** by *Nitrosopumilus piranensis* under oxygen-depletion. Incubation started with a pool of $^{15}NO_2^-$ and $^{44}N_2O$ spiked at 30 h. In figure 1B and 2B averages are shown, while here I, II and III are the individual replicates.

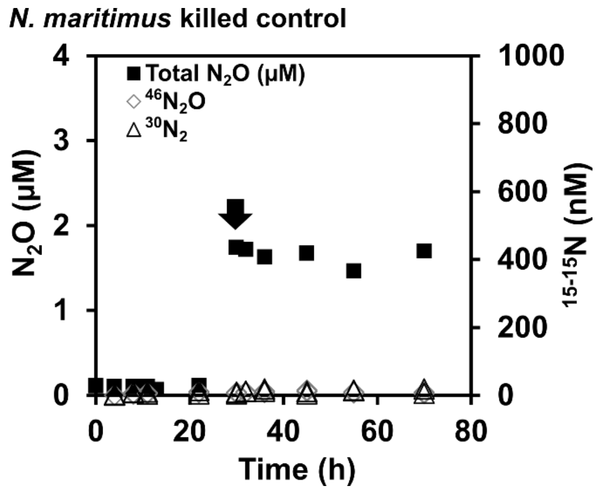

Figure S2. Control testing for abiotic production and consumption of N_2O during the incubation of *N. maritimus*. After the N_2O spike, N_2O concentrations remained stable at the respective new level. Mercuric chloride was added at 0 h.

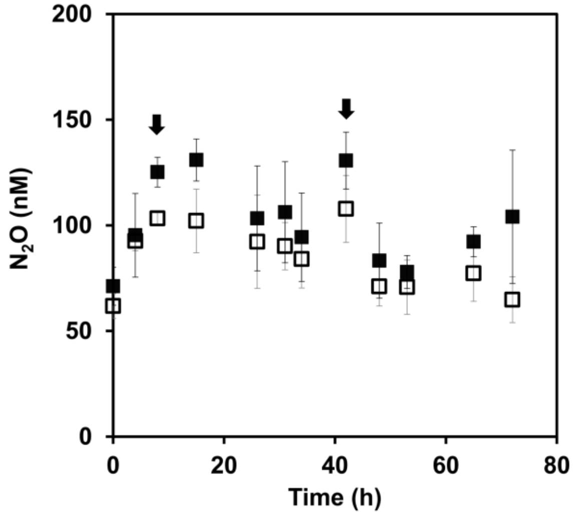

Figure S3. Total N_2O concentration in oxygen-depleted incubation of *N. piranensis* started with a pool of $^{14}NO_2^-$. Black squares represent the average of at least three replicates in which $^{46}N_2O$ was spiked (black arrows represent the time of the spike). Open squares are the control incubations without $^{46}N_2O$ addition. Error bars represent the standard deviation.

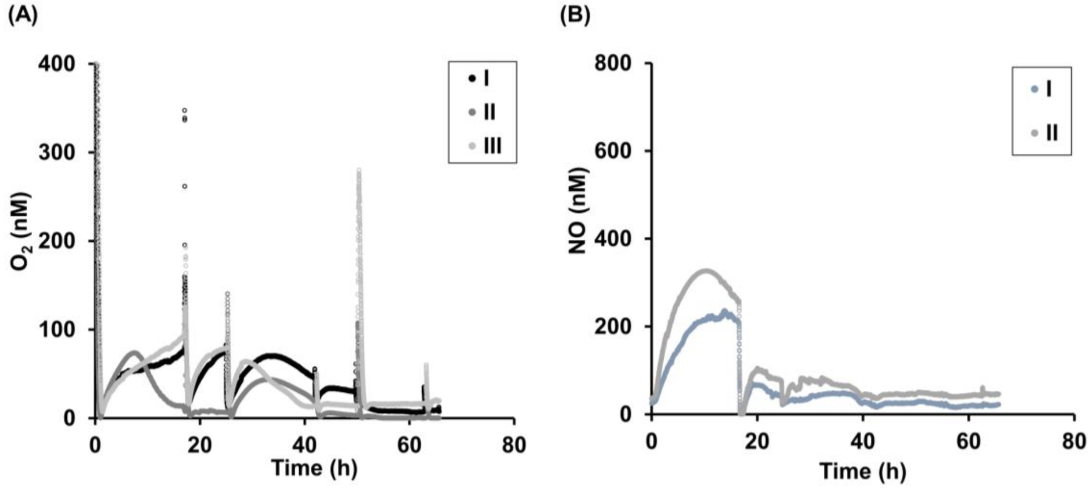

Figure S4. Oxygen and NO accumulation in control incubation of *N. maritimus* with a pool of $^{15}NO_2^-$. **(A)** Oxygen accumulation **(B)** NO concentrations in oxygen-depleted incubation of *N. maritimus* with a pool of $^{15}NO_2^-$. Only two replicates were equipped with an NO microsensor. Each incubation number (I, II, III) represents a replicate of the same incubation.

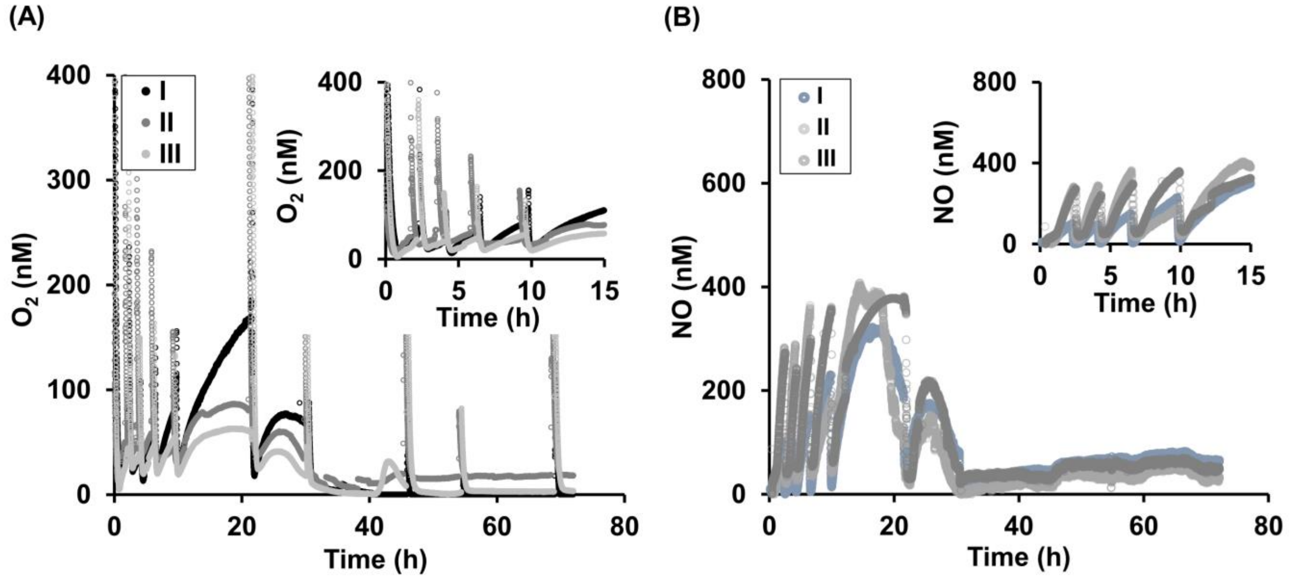

Figure S5. Accumulation of oxygen **(A)** and NO **(B)** in the oxygen-depleted incubation of *N. maritimus* with a pool of $^{15}NO_2^-$ and with a $^{44}N_2O$ spike of 1.2 µM at 30 h. I, II and III are replicates of the same incubation. Inserts show the oxygen or NO accumulation, respectively, within the first 15 h of incubation.

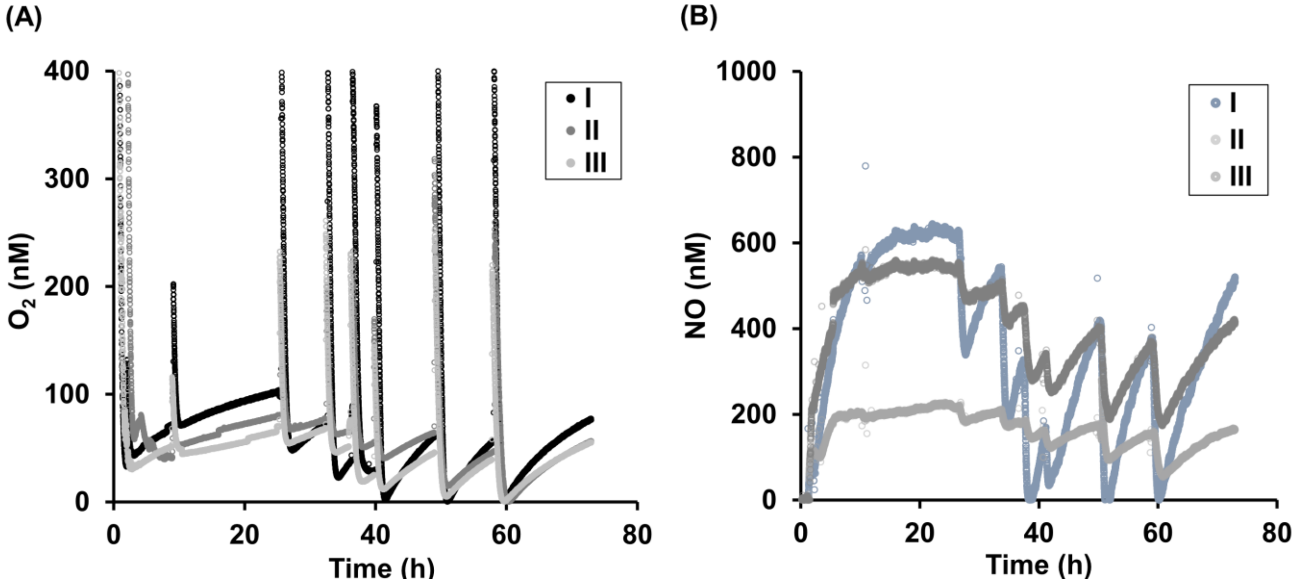

Figure S6. Accumulation of oxygen **(A)** and NO **(B)** in oxygen-depleted incubation of *N. piranensis* with a pool of $^{15}NO_2^-$. This incubation was not used for the N_2O spike experiments reported in the present study. The starting conditions were the same as the other incubations with *N. piranensis* performed in this study (NO_2^- pool and oxygen depletion).

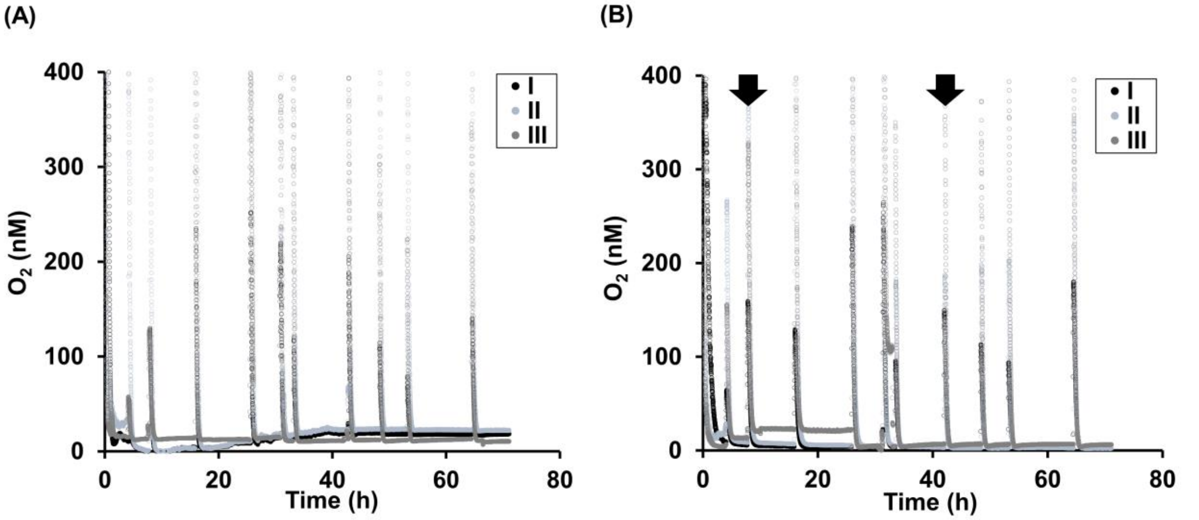

Figure S7. Oxygen dynamics in oxygen-depleted incubation of *N. piranensis* with a pool of $^{14}NO_2^-$ (controls) **(A)** and with a pool of $^{14}NO_2^-$ and spike of $^{46}N_2O$ at 8 h and at 42 h **(B).** Black arrows indicate the N_2O spikes. I, II, and III are replicates of the same treatment.

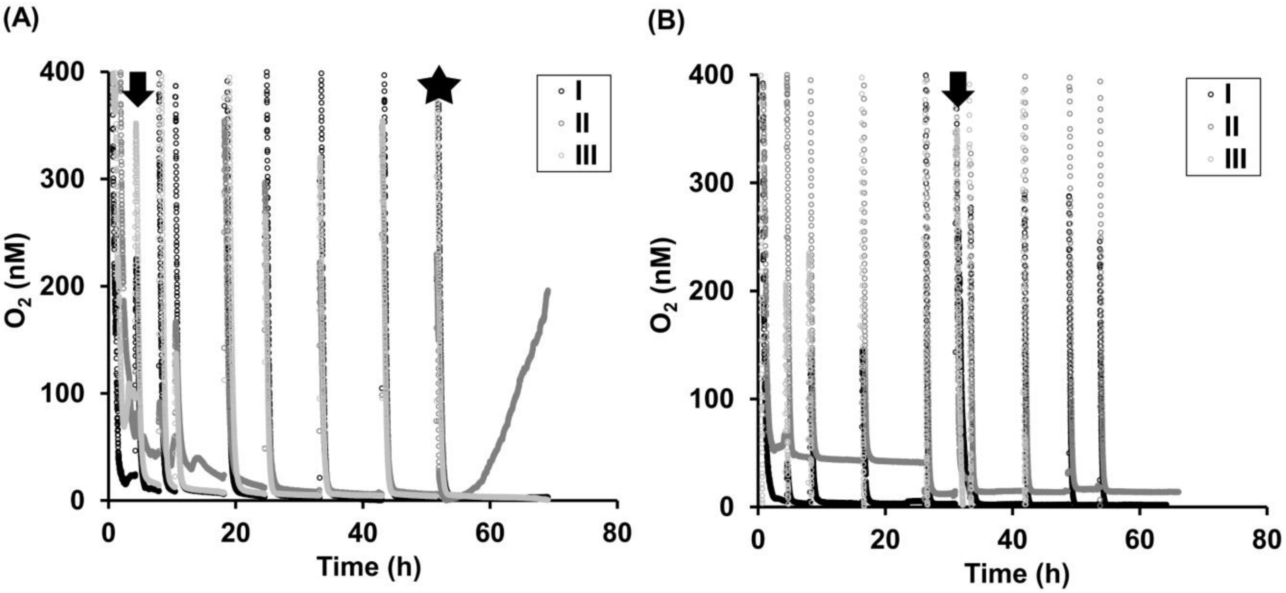

Figure S8. Oxygen dynamics in oxygen-depleted incubation of *N. piranensis* with a pool of $^{15}NO_2^-$ and spikes of $^{46}N_2O$ at 6 h **(A)** and at 30 h **(B).** I, II, and III are replicates of the same treatment. Black star indicates KCN addition to the replicate II of the N_2O spike at 6 h. After KCN was added oxygen accumulation started, in the same way as in the control replicates (Figure 4 D).
